# Near-Infrared Irradiation Nonthermally Induces Long-lasting Vasodilation by Causing Apoptosis of Vascular Smooth Muscle Cells

**Published:** 2011-05-02

**Authors:** Yohei Tanaka, Kiyoshi Matsuo, Shunsuke Yuzuriha

**Affiliations:** Department of Plastic and Reconstructive Surgery Shinshu University School of Medicine, Matsumoto, Japan

## Abstract

**Background:** Moderate sunburn after prolonged sun exposure is thought to cause long-lasting inflammatory vasodilation due to thermal and ultraviolet radiation from the sun. We previously reported that near-infrared irradiation that simulates solar near-infrared can penetrate the skin and nonthermally affect the dermis, superficial muscles, and other tissues. To clarify the possible effect of near-infrared on long-lasting vasodilation, we evaluated how near-infrared affects subcutaneous vascular smooth muscle cells in rats. **Methods:** The central back tissues of rats were irradiated with a near-infrared device that simulates solar radiation, which has specialized contact cooling to avoid thermal effects. The total energy emitted was equivalent to approximately 8.75 hours of sunbathing in North America. Histological evaluation was performed on the subdermal plexus over the panniculus carnosus muscle of the near-infrared–irradiated rats at postirradiation days 7, 30, 60, and 90, and compared with nonirradiated controls. The vascular smooth muscle cells were evaluated by the transferase-mediated dUTP nick-end labeling assay and staining with an anti-smooth muscle actin antibody. **Results:** There was no evidence of inflammation by the increased movement of leucocytes around the dilated vessels in irradiated samples. Near-infrared irradiation induced apoptosis of the vascular smooth muscle cells and significantly induced intense, long-lasting vasodilation of the subdermal plexus at postirradiation day 7. **Conclusions:** Near-infrared irradiation nonthermally induces long-lasting vasodilation by causing apoptosis of vascular smooth muscle cells. Since solar near-infrared radiation nonthermally induces damage of the subcutaneous tissues, exposed skin should be protected with sunscreens that block not only ultraviolet but also near-infrared radiation.

Moderate sunburn after prolonged sun exposure is thought to cause inflammatory vasodilation as a result of thermal and ultraviolet radiation from the sun. However, this inflammatory vasodilation tends to remain longer than expected (Fig 1). Sunlight that reaches the human skin contains solar energy composed of 6.8% ultraviolet light, 38.9% visible light, and 54.3% infrared radiation.^1^ Near-infrared (NIR) is an electromagnetic wave that simultaneously exhibits both wave and particle properties and is strongly absorbed by water, hemoglobin, and myoglobin. We previously reported that NIR irradiation that simulates solar NIR at specific wavelengths with pre- and parallel-irradiational cooling can penetrate the skin and nonthermally affect dermis,^2^^-^4 superficial muscles,^5^,^6^ and other tissues.^7^ To clarify the possible effect of NIR on the long-lasting vasodilation after prolonged sun exposure, we evaluated how NIR affects the subcutaneous vascular smooth muscle cells in rats.

## MATERIALS AND METHODS

### Animals

Thirty male Wistar rats (*Rattus norvegicus albinus*) weighing 360 to 440 g were used. Experiments were performed in a temperature-controlled environment (24° C ± 1.5° C) under a 12-hour light-dark cycle with free access to water and standard rat chow. All animals were treated humanely and in compliance with the recommendations of the local committee on animal care. The study was approved by our institutional ethics committee for animal experiments. Animals were anesthetized with an intra-abdominal dose of sodium pentobarbitone (50 mg/kg, IP) and were killed by intracardiac administration of ketamine (150 mg/kg) upon completion of the experiment.

### NIR irradiation

NIR irradiation was generated using a broadband NIR source (Titan; Cutera, Brisbane, Calif). The device emitted an NIR spectrum ranging from 1100 to 1800 nm and filtering wavelengths between 1400 and 1500 nm, which thereby simulated solar radiation. This procedure allowed us to deliver NIR without the wavelengths that are strongly absorbed by water and hemoglobin, and allowed for the safe delivery of NIR energy deep into the tissue. The horizontal spot size of the irradiation was 10 × 30 mm. Each single shot at 40 J/cm^2^ consists of 4.17-second irradiation periods. To avoid thermal effects, the sapphire contact cooling tip was set to a fixed temperature of 20° C to provide contact cooling. The sapphire block is cooled with fluids using thermoelectric coolers. Cooling fluids are circulated by a pump and cooling system. Pre- and parallel-irradiational cooling of the superficial layers was accomplished using this temperature-controlled sapphire window, which further prevented excessive superficial heating.

Thirty rats were either irradiated (*n* = 20) or not irradiated as a control (*n* = 10). The centers of the dorsal portion (30 × 30 mm) of the irradiated rats were subjected to 3 rounds of irradiation at 40 J/cm^2^ on days 0, 7, and 14 without application of topical anesthesia. We previously reported that 3 rounds of NIR irradiation, which consist of 2 passes at 20 J/cm^2^, are sufficient to induce histological changes in the epidermis of rats, and that higher energies have a greater response and are preferable for effects on deeper tissues.^2^ Correlation to efficacy seemed to be highest with total delivered energy, not per pulse fluence, as lower output, multiple irradiations appeared as equally effective as higher fluence irradiations.^7^ Therefore, we performed NIR irradiation at 40 J/cm^2^. One round of irradiation consisted of 2 passes of NIR irradiation to the area of 10 × 30 mm; thus, 6 passes of NIR irradiation were performed to the center of the dorsal portion. The total energy emitted was equivalent to approximately 8.75 hours of sunbathing in North America.^8^,^9^

### Histological evaluation

Specimens, which included the overlying subcutaneous tissues on the spinous process of the sixth lumbar vertebra, were isolated from the experimental group (5 rats per time point) at 7, 30, 60, and 90 days after the final dose of NIR irradiation (d7, d30, d60, and d90, respectively). Control samples were only isolated at day 0 and day 90 (5 rats per time point). The specimens were fixed in 20% neutral buffered formalin, processed for paraffin embedding, and serially sectioned along the sagittal plane (3- to 4-µm thickness).

Tissue sections were stained with hematoxylin and eosin (H&E), an anti-CD31 antibody to detect the endothelium, and an anti-smooth muscle actin (SMA) antibody to identify the vascular smooth muscle. The transferase-mediated dUTP nick-end labeling (TUNEL) assay was used to stain apoptotic cells.

Cross-sectional areas of the lumens of the subdermal plexus, which were surrounded by the endothelium stained by the anti-CD31 antibody, were calculated for all time points in an area 0.2 mm high × 3 mm wide on the panniculus carnosus over the middle of the spinous process.

Images were scanned and quantified in 5 representative fields per section and subsequently averaged to obtain a final score. The sections were photographed under an Olympus BX50 microscope (Olympus, Tokyo, Japan). The digital photographs were processed using Adobe Photoshop (Adobe, San Jose, Calif).

### Statistical analyses

The differences between groups at each time point were examined for statistical significance using the Mann-Whitney *U* test. *P* < .05 was set as a cutoff for statistical significance.

## RESULTS

There was no evidence of acute or chronic inflammation by the increased movement of leukocytes or fibrocytes around the dilated vessels at any time point in the irradiated samples.

Apoptotic cells were detected in the layer of vascular smooth muscle cells identified by anti-SMA staining at d7, but were not detected at d30, d60, and d90, or in the controls at d0 and d90.

**Figure 2:**
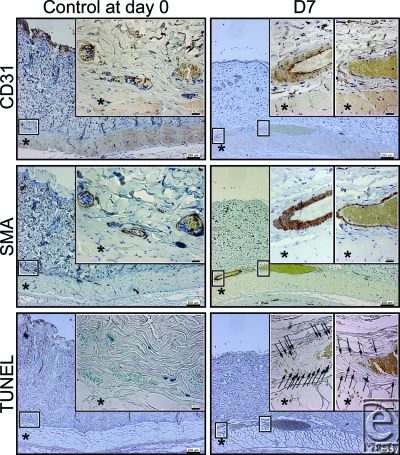
The histology of control rats at day 0 (left column) and near-infrared (NIR)–irradiated rats at 7 days (d7) after the final dose of irradiation (right column). Skin and panniculus carnosus were evaluated by immunohistochemical staining with an anti-CD31 antibody (first row), anti-smooth muscle actin (SMA) antibody (second row), and TUNEL (third row). A representative section of the subdermal plexus enclosed in the smaller box of the corresponding size is enlarged in the larger box of the same size. CD31-positive cells, SMA-positive cells, and TUNEL-positive cells are stained brown. Representative TUNEL-positive cells are indicated by black arrows. The asterisk (*) indicates the panniculus carnosus. Scale bars = 200 µm (magnification: 40×). Insets: scale bars = 20 µm (magnification: 400×).

**Figure 3:**
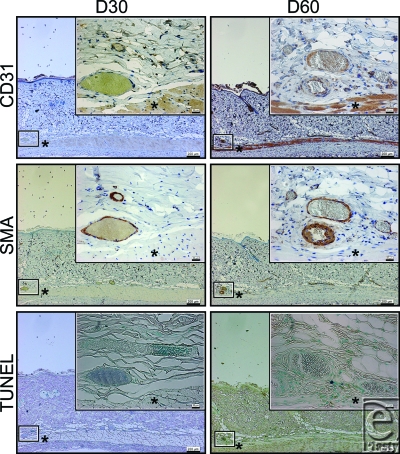
Histology of NIR irradiated rats at 30 d (left column) and 60 d (right column). Skin and panniculus carnosus were evaluated by immunohistochemical staining with an anti-CD31 antibody (first row), anti-SMA antibody (second row), and TUNEL (third row). A representative section of the subdermal plexus enclosed in the smaller box of the corresponding size is enlarged in the larger box of the same size. The asterisk (*) indicates the panniculus carnosus. Scale bars = 200 µm (magnification: 40×). Insets: scale bars = 20 µm (magnification: 400×).

**Figure 4:**
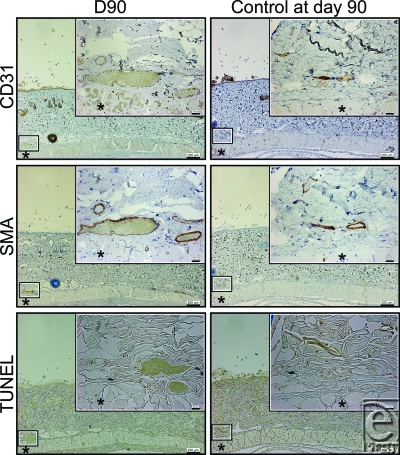
Histology of NIR-irradiated rats at day 90 (left column) and nonirradiated control rats at day 90 (right column). Skin and panniculus carnosus were evaluated by immunohistochemical staining with an anti-CD31 antibody (first row), anti-SMA antibody (second row), and TUNEL (third row). A representative section of the subdermal blood plexus enclosed in the smaller box of the corresponding size is enlarged in the larger box of the same size. The asterisk (*) indicates the panniculus carnosus. Scale bars = 200 µm (magnification: 40×). Insets: scale bars = 20 µm (magnification: 400×).

**Figure 5:**
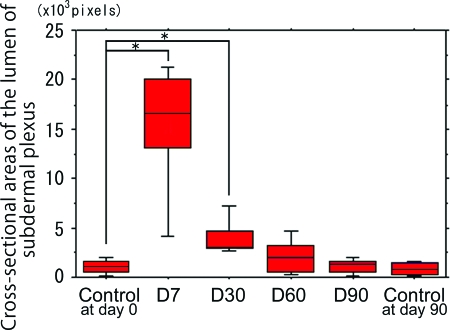
Mean changes in the cross-sectional areas of the lumen of the subdermal plexus at days 0 and 90 (controls), as well as days 7, 30, 60, and 90 after the final dose of NIR irradiation (d7, d30, d60, and d90, respectively). Data represent the means ± SD. Significant differences are indicated (*P < .05).

Cross-sectional areas of the lumen of the subdermal plexus were abruptly dilated by the NIR irradiation at d7 and subsequently showed a gradual shrinkage thereafter (Figs 2-5). Statistically significant increases in the cross-sectional areas of the lumen of the subdermal plexus were observed at d7 and d30 compared with nonirradiated controls at d0 and d90, respectively (*P* < .05) (Fig 5). No statistically significant increase was observed at d60 or d90 compared with the nonirradiated controls at the same time points (*P* = .3472 and .6015, respectively). No changes were observed between the nonirradiated controls at d0 and at d90 (*P* = .7540) (Fig 5).

## DISCUSSION

Near-infrared irradiation has previously been reported to induce strand breaks and cell death by apoptosis.^5^,^7^,^10^ TUNEL staining identifies DNA fragmentation and therefore stains cells undergoing either apoptosis or necrosis. In this study, we did not detect any evidence of increased movement of leukocytes or fibrocytes. In addition, apoptotic cells detected by TUNEL staining were observed in the layer of vascular smooth muscle identified by anti-SMA staining at postirradiation day 7 (Fig 2). Therefore, these results suggest that NIR irradiation simulated solar radiation nonthermally induced long-lasting vasodilation not by inflammation, but by apoptosis of the vascular smooth muscle cells at postirradiation day 7.

Acute vasodilation is one of the classical signs of acute inflammation that increases blood flow for the acute inflammatory process. Therefore, acute vasodilation that occurs immediately after the sun exposure may result from acute inflammation due to thermal and ultraviolet radiation from the sun. Since the healing period in rats is approximately 2 to 3 times shorter than that in humans,^3^ the long-lasting vasodilation at postirradiation day 7 may not to be due to acute vasodilation. Since water and hemoglobin can absorb NIR, long-lasting vasodilation may allow for the accumulation of blood in the dilated vessels to protect the subcutaneous tissues from NIR irradiation.

Based on these findings, there are several diseases reported that may relate to long-lasting vasodilation due to chronic NIR radiation, such as rosacea and erythema ab igne. Rosacea is a chronic cutaneous disorder characterized by centrofacial persisting erythema, telangiectases, papules, pustules, edema, and ocular involvement. Although rosacea is one of the most common skin disorders, its pathogenesis remains unclear and controversial. Rosacea affects all races; however, it has been generally accepted that the disease is more common in whites and fair-skinned populations.^11^ Near-infrared radiation due to long-term sun exposure should be considered as a critical factor in the development and aggravation of rosacea, since the distribution of erythema is most prominent on the facial convexities.^12^ The occurrence of these telangiectases was shown to increase with increased age, increased sunbathing, and poor pigmentation ability.^11^ On the contrary, erythema ab igne,^13^ which means “redness from fire,” was first observed on the legs of women who sat close to coal stoves. Long-term exposure to moderate infrared radiation from various heat sources is thought to thermally induce reticulated erythema and result in histopathologic changes similar to those seen in solar-damaged skin.

Near-infrared irradiation is known to be of therapeutic benefit in the treatment of musculoskeletal disorders and healing of indolent wounds^14^,^15^ and appears to induce nonthermal, long-lasting vasodilation for an increase in blood circulation. Despite the widespread therapeutic potential of NIR, the mechanisms responsible for the therapeutic actions of photobiomodulation with NIR have not been elucidated in detail. Photobiomodulation with NIR increases mitochondrial metabolism,^16^^-^19 facilitates wound healing, and promotes angiogenesis in skin,^20^ bone,^21^ nerve,^22^ and skeletal muscle.^23^,^24^ In addition, NIR irradiation is potentially applicable for the treatment of vasospasm and flap delay in the field of plastic and reconstructive surgery.

Regarding the mechanism of the NIR irradiation-mediated induction of apoptosis of vascular smooth muscle cells, NIR irradiation may injure the myoglobin of smooth muscles cells and result in apoptosis. However, further studies are required to confirm this hypothesis.

## CONCLUSIONS

Near-infrared irradiation nonthermally induces long-lasting vasodilation by causing apoptosis of vascular smooth muscle cells. Although sunburn is thought to occur when skin has been burned by thermal and ultraviolet radiation, solar NIR radiation nonthermally affects the subcutaneous tissues, including the dermis, subdermal plexus, and superficial muscles. Therefore, exposed skin might be better protected with sunscreens that block not only ultraviolet but also NIR radiation.

## Acknowledgments

We thank Ikuo Matsuyama for the histological staining.

## Figures and Tables

**Figure 1:**
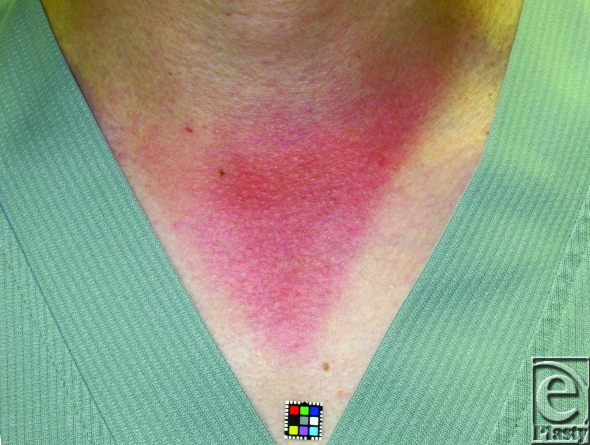
Moderate sunburn 3 days after an 8-h exposure to the sun.
